# PEG 400/Cerium Ammonium Nitrate Combined with Microwave-Assisted Synthesis for Rapid Access to Beta-Amino Ketones. An Easy-to-Use Protocol for Discovering New Hit Compounds

**DOI:** 10.3390/molecules23040775

**Published:** 2018-03-28

**Authors:** Giacomo Rossino, Maria Valeria Raimondi, Marta Rui, Marcello Di Giacomo, Daniela Rossi, Simona Collina

**Affiliations:** 1 Drug Sciences Department, Medicinal Chemistry and Pharmaceutical Technology Section, University of Pavia, 27100 Pavia, Italy; giacomo.rossino01@universitadipavia.it (G.R.); marta.rui01@universitadipavia.it (M.R.); marcello.digiacomo@unipv.it (M.D.G.); daniela.rossi@unipv.it (D.R.); 2 Department of Biological, Chemical and Pharmaceutical Sciences and Technologies, Medicinal Chemistry and Pharmaceutical Technologies Section, University of Palermo, 90100 Palermo, Italy; mariavaleria.raimondi@unipa.it

**Keywords:** Mannich reaction, β-aminoketones, microwave-assisted organic synthesis, polymer-assisted solution phase synthesis, solid phase extraction, drug discovery

## Abstract

Compound libraries are important requirement in target-based drug discovery. In the present work, a small focused compound library based on β-aminoketone scaffold has been prepared combining microwave-assisted organic synthesis (MAOS) with polymer-assisted solution phase synthesis (PASPS) and replacing reaction workup standard purification procedures with solid phase extraction (SPE). Specifically, the effects of solvent, such as dioxane, dimethylformamide (DMF), polyethylene glycol 400 (PEG 400), temperature, irradiation time, stoichiometric ratio of reagents, and catalysts (HCl, acetic acid, cerium ammonium nitrate (CAN)) were investigated to maximize both conversion and yield. The optimized protocol generally afforded the desired products in satisfying yields and purities. The designed library is a part of our current research on sigma 1 receptor modulators, a valuable tool for the identification of novel potential hit compounds.

## 1. Introduction

Identifying hit compounds is the first step in the complex drug-discovery process, and the degree of structural diversity is an important element, enhancing the rate of success in finding a potential lead candidate. In this context, β-amino carbonyl compounds represent a class of important pharmacophores and useful building blocks for the synthesis of diverse classes of biologically active molecules [1,2].

**Figure 1:**
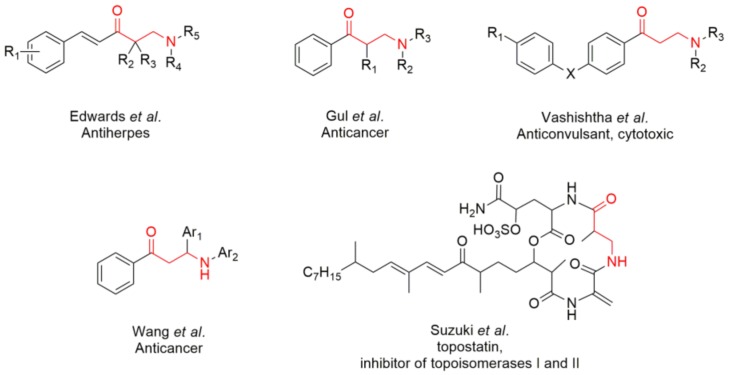
Some important β-amino ketones, both synthetic and natural, and their biological properties.

Numerous β-amino ketones and their analogues exhibit potent activity of great interest in medicinal chemistry, such as anti-inflammatory [3,4], antibacterial [5,6], antiviral [7], antifungal [6,8], analgesic [9], and anticancer activity [10,11,12,13], to cite just a few examples (Figure 1). Moreover, β-amino acids are found in some important bioactive natural compounds and are widely employed in the preparation of peptide-based drugs [14,15,16,17] (Figure 1). No less important, β-amino ketones can be key intermediates for the synthesis of pharmaceutically relevant compounds [18,19].

**Figure 2:**
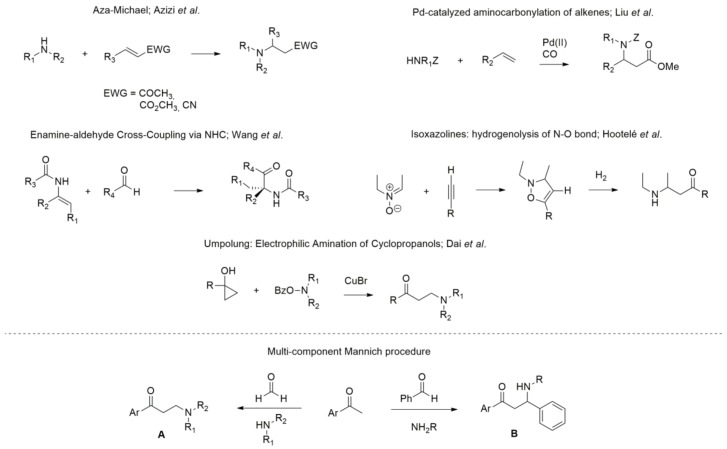
Alternative synthetic routes for accessing β-amino carbonyl compounds.

While many synthetic strategies to achieve β-amino carbonyl compounds can be found in the recent literature (Figure 2), such as aza-Michael reaction [20], enamine-aldehyde cross-coupling via *N*-heterocyclic carbenes [21], copper-catalyzed electrophilic amination of cyclopropanols [22], Pd-catalyzed aminocarbonylation of alkenes [23], and hydrogenolysis of isoxazolines [24], the Mannich multicomponent reaction (Figure 2) remains the most used procedure [2,25], and many improvements to and implementations of the original protocol have been studied [19,26].

In particular, the three-component one-pot Mannich reaction allows the formation of β-amino ketones, presenting general structure A or B (Figure 2) with great structural variability, depending on the amine and aldehyde employed [27,28].

In light of these considerations and as part of our ongoing research, we herein focus on the development of an efficient protocol based on the three-component one-pot Mannich reaction for the preparation of a β-amino ketone small library endowed with general formula A (Figure 2), consisting of a tertiary amine bridged to an aromatic ring by a propylenic chain. The final aim is to discover new potential sigma receptor (SR) modulators [29,30,31,32]. We set up an efficient, clean, quick, and scalable protocol based on microwave-assisted organic synthesis (MAOS), using cerium ammonium nitrate (CAN) as a catalyst and polyethylene glycol 400 (PEG 400) as a solvent, combined with polymer-assisted solid phase synthesis (PASPS). Purification of final compounds occurred by solid phase extraction (SPE). Overall, our strategy led us to obtain the desired β-amino ketones efficiently and quickly.

## 2. Results and Discussion

**Figure 3:**
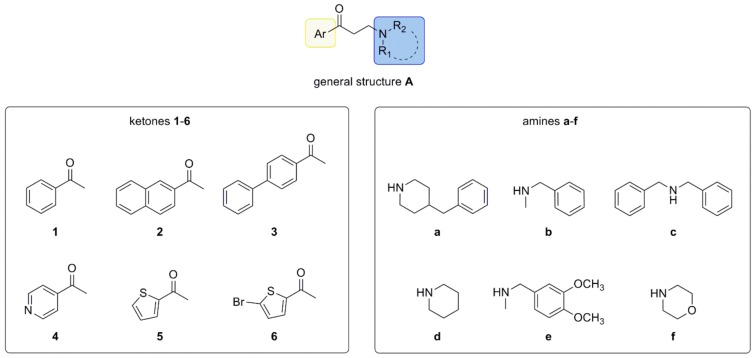
Designed library.

Through this procedure, a small focused library of 36 β-amino ketones derived from the coupling of aryl-ketones **1**–**6** with amines **a**–**f** (Figure 3) was prepared. Relying on our long experience in the SR field, both building blocks were selected by taking into account the state-of the-art structure activity relationship (SAR) of SR ligands [33,34]. We exploited aromatic or heterocyclic methyl-ketones (**1**–**6**) and cyclic (**a**, **d**, **f**) or benzyl acyclic (**b**, **c**, **e**) secondary amines (Figure 3).

### 2.1. Setup and Optimization of Synthetic Protocol

**Figure 4:**
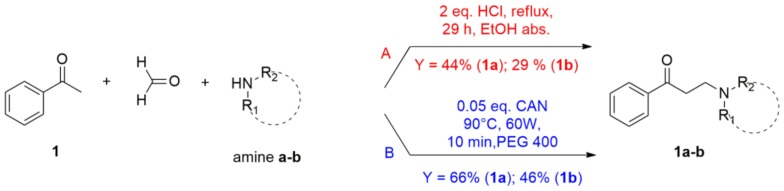
Comparison between (A) traditional and (B) new protocol.

According to data in the literature concerning the different reactivities of secondary amines related to their structures and experimental conditions in the Mannich reaction [25,35], we set up a novel protocol using the cyclic and acyclic amines **a** and **b** as “building block” models. First, compounds **1a** and **1b** were synthesized with conventional heating, applying an existing protocol (Figure 4, condition A), and were properly purified [36]. Molar extinction coefficients of acetophenones **1**, **1a**, and **1b** were determined (1.265 × 10^4^, 6.327 × 10^3^, and 6.703 × 10^3^ L·mol^−1^·cm^−1^, respectively) and high performance liquid chromatography-ultraviolet-photodiode array detector (HPLC-UV-PAD) methods were devecloped to determine the percentage of conversion and purity of new compounds. Afterwards, based on our own experience, we set up a microwave-assisted synthetic protocol (Figure 4) to obtain our β-amino ketones **1**–**6**, **a**–**f**. Of note, MAOS has already been successfully employed in Mannich reactions [37,38]. Microwave oven parameters (i.e., temperature, irradiation power, and time) were explored and different solvents, such as dioxane, dimethylformamide (DMF), tetrahydrofuran (THF), methanol (MeOH), ethanol (EtOH) tested. Temperature and irradiation power varied from 35 °C to 200 °C and from 60 W to 200 W, respectively, as did irradiation time. Lastly, both type and amount of protic acidic additive were evaluated (HCl, HBF_4_, HClO_4_, acetic acid). Unfortunately, no satisfying results were obtained. Therefore, we considered the use of ceric ammonium nitrate (CAN) as a catalyst in PEG 400, as it had already been used in a three-component Mannich reaction to access β-amino ketones of general structure B (Figure 2) under conventional heating [39]. Accordingly, we employed this catalyst/solvent combination in our microwave-assisted protocol to access the designed compounds of general structure A (Figure 2). Compounds **1a** and **1b** were obtained under microwave irradiation (60 W, 90 °C for 10 min) using 5% mol of CAN in PEG 400. The HPLC analysis (see Appendix A) showed that the reaction was clean and quick, affording the desired products with 80% conversion. Interestingly, using hydrochloride amines as reagents led to the best results. A schematic comparison between the old and new protocols (path A and path B, respectively) is shown in Figure 4.

With these promising results, we moved forward to determine the effect of stoichiometric ratio of reagents on both conversion percentage and crude purity. Results are reported in Table 1 and Table 2. Reactions conducted with excess amine (entries 2–4 and 10–13) led to high conversion percentages, even if the products had lower purities. An opposite trend was observed using an excess of ketone (entries 5–8 and 14–17). Accordingly, conditions of entries 6 and 15 (i.e., 2 equivalents (eq.) of ketone and 1 eq. of amine) were considered the best compromise and were extended to the other substrate for preparation of the whole library.

### 2.2. MW-Assisted Library Synthesis

The optimized protocol was then employed to synthesize the small focused library. The following table reports the yields and purities of each compound.

The developed protocol allowed production of the desired compounds (Table 3) with the exception of dibenzylaminic (**1**–**6c**) and 4 acetyl-pyridinic (**4a**–**f**) compounds (Figure 3). Of note, cyclic amines (**a**, **d**, **f**) were well tolerated in this protocol; in particular, piperidines were the most versatile reagents, since they were able to react with the ketone counterpart, offering products in satisfying yields (Table 3). Relying on the molecules endowed with an acyclic amine, different reactivity was seen: *N*-benzylmethylamine > 3,4-dimethoxy-*N*-methylbenzylamine > dibenzylamine. The failure in the reaction involving dibenzylamine may be attributed to its low basicity, which may slow down the formation of the intermediate imine resulting from the reaction with formaldehyde. This result is not surprising. Indeed, it has been shown that the reactivity in the Mannich reaction is strictly related to the amine structure, and within a homologous series the reactivity may be different. An emblematic example is diethylamine, which is unable to be transformed into β-amino ketone, whereas the superior and inferior amine analogues react efficiently to give access to the desired products [35]. Regarding the ketone building block, the procedure was successfully applied to aromatic ketones, presenting benzene and thienyl nucleus, whereas no product was observed when ketone **4**, bearing a pyridine moiety, was used. This behavior can be explained by considering the basic properties of the pyridine ring, which reduced the reactivity of methyl ketone **4**. We performed additional model reactions on ketone **4** under traditional conditions at high temperature and for long times to force the reaction. Again, the desired products (compounds **4a** and **4b**) were not isolated, supporting our hypothesis that the low reactivity of 4 acetyl-pyridine compromised the reaction outcome.

## 3. Materials and Methods

Reactions performed under conventional heating were monitored by thin layer chromatography (TLC) with Fluka silica gel 60 F254 (Merck KGaA, Darmstadt, Germany) and purified by automatic flash chromatography with CombiFlash**^®^**RF (AlfaTech, Teledyne Isco, Inc., Genoa, Italy).

All reactions conducted under microwave irradiation were performed in a microwave mono-mode oven specifically for organic synthesis (Discover**^®^** LabMate instrument, CEM Corporation, Matthews, NC, USA). The obtained products were purified with Bond Elut SCX**^®^** cartridge (Varian, Walnut Creek, CA, USA) and silica gel SPE cartridge (Varian, Walnut Creek, CA, USA).

**Figure A1:**
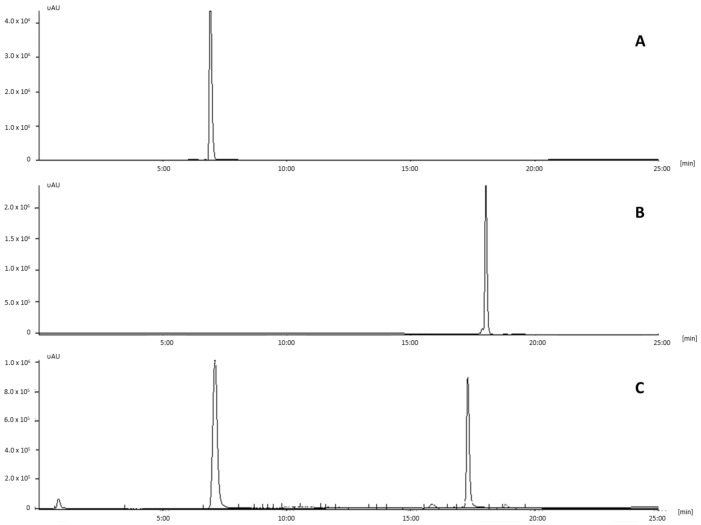
(**A**) Acetophenone (RT: 6.8 min); (**B**) compound **1a** (RT: 16 min); (**C**) reaction mixture (MW) acetophenone (2.0 eq.), 4-benzylpiperidine hydrochloride (1 eq.), paraformaldehyde (1 eq.).

**Figure A2:**
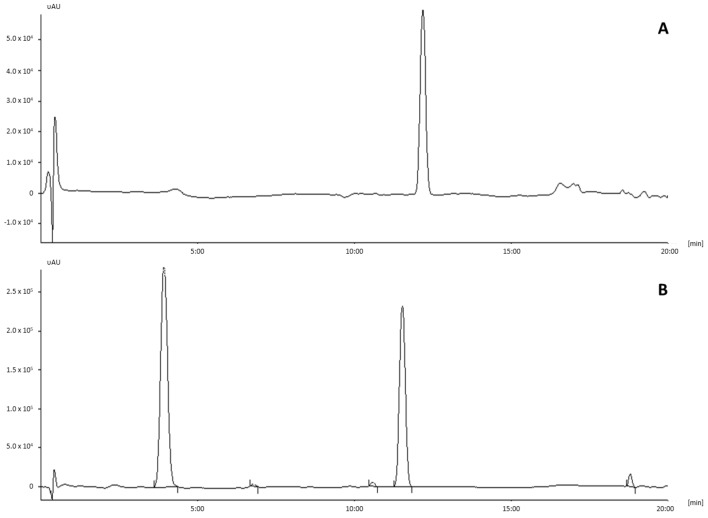
(**A**) Compound **1b** (RT: 12 min); (**B**) reaction mixture (MW) acetophenone (2.0 eq.), *N*-benzylmethylamine hydrochloride (1 eq.), paraformaldehyde (1 eq.).

UV spectra were recorded on a LAMBDA™ 25 UV/VIS spectrometer (Perkin Elmer Inc., Waltham, MA, USA). HPLC analyses were carried out on a Jasco HPLC system (Jasco Europe S.r.l., Cremella, Italy), consisting of a pump model PU 1580, a Reodyne 7125 injector (20 μL sample loop), and an MD-1510 diode array detector, combined with a Spectra AS3000 autosampler. Experimental data were acquired and interpreted with Borwin PDA and Borwin chromatograph software 1.5. Reversed-phase HPLC analyses were carried out at room temperature on an XTerra RP18 column (3.5 μm, 4.6 × 50 mm) (Waters, Milford, MA, USA) and a Hypersil ODS RP18 column (3 μm, 4.6 × 100 mm) (VWR, Milano, Italy). The mobile phase was phosphate buffer (pH 7.8) added with acetonitrile as organic modifier; the analysis was carried out using gradient elution (see Table A1 and Table A2, Figure A1 and Figure A2 in Appendix A).

Electrospray ionization LC-MS analyses were performed with a single quadrupole AQA ThermoQuest Finnigan (ThermoFinnigan, San Jose, CA, USA) or a Waters Micromass ZQ2000 (Waters, Milford, MA, USA), employing an XBridge C8 column (3.5 μm, 4.6 × 50 mm) (Waters, Milford, MA, USA).

^1^H-NMR spectra were registered with a Brüker ARX 300 (300 MHz) (Bruker Daltonics, Billerica, MA, USA). Chemical shifts are reported in parts per million (δ) downfield from tetramethylsilane (TMS) as internal standard.

### 3.1. General Procedure for the Synthesis of β-Aminoketone ***1a*** and ***1b*** under Conventional Heating (Method A)

A solution of acetophenone (1.0 eq.), amine (2.0 eq.), paraformaldehyde (2.0 eq.), and HCl (2.0 eq.) in absolute ethanol (2.25 mL) was refluxed for 24 h in N_2_ atmosphere under magnetic stirring. Then, the reaction mixture was evaporated under reduced pressure and the residue purified by automated flash chromatography (CombiFlash^®^RF) using a mixture of 80:20 hexane:diethyl ether, 0.1 NH_3_/MeOH as eluent, and silica gel RediSep column (12 g) (particle size: 35–70 μm).

### 3.2. General Procedure for the Synthesis of β-Aminoketones ***1a***–***6f*** under Microwave Heating (Method B)

A mixture of ketone (2.0 eq.), amine hydrochloride (1 eq.), paraformaldehyde (1 eq.), and CAN (0.05 eq.) in PEG 400 (0.8 mL) was irradiated with a microwave power of 60 watts at 90 °C for 10 min. The reaction workup was performed as follows: the mixture was quenched in 2 M NaOH, then the solid was collected by centrifugation, dissolved in methanol or dichloromethane (depending on the solubility of the compound), and purified using SCX cartridge, eluting with a solution of 0.3 M NH_3_/MeOH in dichloromethane to remove the excess ketone. Finally, the product was isolated using silica gel SPE cartridge, eluting with dichloromethane to remove the nonreacted amine. Then the organic phase was evaporated to dryness.

### 3.3. Analytical Data of Prepared Compounds

*3-(4-Benzylpiperidin-1-yl)-1-phenylpropan-1-one* (**1a**). Yield: 44% (method A), 72% (method B). Yellow oil; ^1^H-NMR (300 MHz, CDCl_3_) (ppm): 1.62 (br s, 2H), 1.74 (d, 3H), 2.26 (br s, 2H), 2.58 (d, 2H), 2.95–3.26 (m, 4H), 3.43 (t, 2H), 7.10–7.25 (m, 3H), 7.25–7.33 (m, 2H), 7.43–7.53 (m, 2H), 7.53–7.63 (m, 1H), 7.92–8.03 (m, 2H); LC-MS: Purity 98%; RT 4.53 min. MH^+^ 308.14 [40].

*3-[Benzyl(methyl)amino]-1-phenylpropan-1-one* (**1b**). Yield: 70%; Yellow oil; ^1^H-NMR (300 MHz, CDCl_3_) (ppm): 2.34 (s, 3H), 3.00 (t, 2H), 3.29 (t, 2H), 3.67 (br s, 2H), 7.28–7.32 (m, 1H), 7.32–7.39 (m, 4H), 7.42–7.50 (m, 2H), 7.53–7.61 (m, 1H), 7.92–7.88 (m, 2H); LC-MS: Purity 86%; RT 3.84 min. MH^+^ 254.11 [41].

*1-Phenyl-3-(piperidin-1-yl)propan-1-one* (**1d**). Yield: 58%; ^1^H-NMR (300 MHz, CDCl_3_) (ppm): 1.49–1.57 (m, 2H), 1.62–1.79 (m, 4H), 2.54–2.71 (m, 4H), 2.94 (t, 2H), 3.34 (t, 2H), 7.41–7.52 (m, 2H), 7.53–7.63 (m, 1H), 7.94–8.01 (m, 2H); LC-MS: Purity 75%; RT 3.12 min. MH^+^ 218.16 [42].

*3-[(3,4-Dimethoxybenzyl)(methyl)amino]-1-phenylpropan-1-one* (**1e**). Yield: 33%; Yellow oil; ^1^H-NMR (300 MHz, CDCl_3_) (ppm): 2.34 (s, 3H), 2.94–3.06 (m, 2H), 3.21–3.33 (m, 2H), 3.71 (s, 2H), 3.88 (s, 6H), 6.65–6.77 (m, 2H), 6.77–6.87 (m, 2H), 7.42–7.53 (m, 2H), 7.53–7.62 (m, 1H), 7.91–8.05 (m, 1H); LC-MS: Purity 66%; RT 3.79 min. MH^+^ 314.12.

*3-(Morpholin-4-yl)-1-phenylpropan-1-one* (**1f**). LC-MS: Purity 5%; RT 2.73 min. MH^+^ 220.11 [41]

*3-(4-Benzylpiperidin-1-yl)-1-(naphthalen-2-yl)propan-1-one* (**2a**). Yield: 32%; Yellow oil; ^1^H-NMR (300 MHz, CDCl_3_) (ppm): 1.52–1.88 (m, 5H), 2.19–2.48 (m, 2H), 2.60 (d, 2H), 3.04–3.35 (m, 4H), 3.46–3.76 (m, 2H), 7.12–7.24 (m, 3H), 7.28–7.36 (m, 2H), 7.47–7.70 (m, 2H), 7.84–7.94 (m, 2H), 7.95–8.08 (m, 2H), 8.54 (s, 1H); LC-MS: Purity 69%; RT 4.99 min. MH^+^ 358.09 [40].

*3-[Benzyl(methyl)amino]-1-(naphthalen-2-yl)propan-1-one* (**2b**). Yield: 34%; ^1^H-NMR (300 MHz, CDCl_3_) (ppm): 2.38 (s, 3H), 3.06 (t, 2H), 3.43 (t, 2H), 3.71 (s, 2H), 7.30–7.41 (m, 5H), 7.52–7.66 (m, 2H), 7.85–7.93 (m, 2H), 7.97 (d, 1H), 8.02 (dd, 1H), 8.47 (s, 1H); LC-MS: Purity 81%; RT 4.51 min. MH^+^ 304.11 [41].

*1-(Naphthalen-2-yl)-3-(piperidin-1-yl)propan-1-one* (**2d**). Yield: 50%; ^1^H-NMR (300 MHz, CDCl_3_) (ppm): 1.44–1.63 (m, 2H), 1.72–1.91 (m, 4H), 2.60–2.88 (m, 4H), 3.11 (t, 2H), 3.57 (br t, 2H), 7.46–7.68 (m, 2H), 7.83–8.11 (m, 4H), 8.53 (br s, 1H); LC-MS: Purity 75%; RT 4.04 min. MH^+^ 268.14 [42].

*3-((3,4-Dimethoxybenzyl)(methyl)amino)-1-(naphthalen-2-yl)propan-1-one* (**2e**). Yield: 38%; Yellow oil; ^1^H-NMR (300 MHz, CDCl_3_) (ppm): 2.32 (s, 3H), 2.98 (t, 2H), 3.34 (t, 2H), 3.60 (s, 2H), 3.85 (s, 6H), 6.8 (m, 3H), 7.70 (m, 2H), 7.98–8.15 (m, 4H), 8.50 (s, 1H); LC-MS: Purity 54%; RT 4.41 min. MH^+^ 364.08.

*3-(Morpholin-4-yl)-1-(naphthalen-2-yl)propan-1-one* (**2f**). Yield: 46%; ^1^H-NMR (300 MHz, CDCl_3_) (ppm): 2.76 (br s, 4H), 3.10 (br t, 2H), 3.51 (br t, 2H), 3.86 (br t, 4H), 7.53–7.66 (m, 2H), 7.86–7.94 (m, 2H), 7.96–8.01 (m, 1H), 8.04 (dd, 1H), 8.51 (br s, 1H); LC-MS: Purity 71%; RT 3.78 min. MH^+^ 270.09 [42].

*3-(4-Benzylpiperidin-1-yl)-1-(biphenyl-4-yl)propan-1-one* (**3a**). Yield: 75%; ^1^H-NMR (300 MHz, CDCl_3_) (ppm): 1.67–1.84 (m, 5H), 2.58 (br d, 2H), 3.18–3.37 (m, 4H), 3.53–3.71 (m, 4H), 7.09–7.17 (m, 2H), 7.19–7.24 (m, 1H), 7.28–7.32 (m, 1H), 7.37–7.52 (m, 4H), 7.60–7.66 (m, 2H), 7.67–7.74 (m, 2H), 8.03–8.09 (m, 2H); LC-MS: Purity 75%; RT 5.21 min. MH^+^ 384.12.

*3-[Benzyl(methyl)amino]-1-(biphenyl-4-yl)propan-1-one* (**3b**). Yield: 55%; ^1^H-NMR (300 MHz, CDCl_3_) (ppm): 2.40 (s, 3H), 3.02–3.15 (m, 2H), 3.39 (t, 2H), 3.75 (br s, 2H), 7.28–7.44 (m, 6H), 7.45–7.53 (m, 2H), 7.60–7.66 (m, 2H), 7.66–7.73 (m, 2H), 7.99–8.07 (m, 2H); LC-MS: Purity 67%; RT 4.83 min. MH^+^ 330.11.

*1-(Biphenyl-4-yl)-3-(piperidin-1-yl)propan-1-one* (**3d**). Yield: 50%; ^1^H-NMR (300 MHz, CDCl_3_) (ppm): 1.51–1.66 (m, 2H), 1.72–1.86 (m, 4H), 2.66–2.78 (m, 4H), 3.06 (t, 2H), 3.46 (br t, 2H), 7.38–7.52 (m, 3H), 7.61–7.66 (m, 2H), 7.67–7.73 (m, 2H), 8.03–8.10 (m, 2H); LC-MS: Purity 73%; RT 4.38 min. MH^+^ 294.16.

*3-[(3,4-Dimethoxybenzyl)(methyl)amino]-1-(biphenyl-4-yl)propan-1-one* (**3e**). LC-MS: Purity 5%; RT 4.73 min. MH^+^ 390.03.

*1-(Biphenyl-4-yl)-3-(morpholin-4-yl)propan-1-one* (**3f**). Yield: 50%; ^1^H-NMR (300 MHz, CDCl_3_) (ppm): 2.70–2.86 (m, 4H), 3.04–3.16 (m, 2H), 3.38–3.50 (m, 2H), 3.84–3.93 (m, 4H), 7.39–7.53 (m, 3H), 7.61–7.67 (m, 2H), 7.68–7.73 (m, 2H), 8.03–8.09 (m, 2H); LC-MS: Purity 61%; RT 4.18 min. MH^+^ 296.11 [40].

*3-(4-Benzylpiperidin-1-yl)-1-(thiophen-2-yl)propan-1-one* (**5a**). Yield: 63%; ^1^H-NMR (300 MHz, CDCl_3_) (ppm): 1.37–1.77 (m, 5H), 2.12 (t, 2H), 2.57 (d, 2H), 2.92 (t, 2H), 2.98–3.08 (m, 2H), 3.23 (t, 2H), 7.12–7.17 (m, 3H), 7.19–7.28 (m, 2H), 7.28–7.32 (m, 1H), 7.63–7.67 (m, 1H), 7.77 (dd, 1H); LC-MS: Purity 95%; RT 4.40 min. MH^+^ 314.05 [43].

*3-[Benzyl(methyl)amino]-1-(thiophen-2-yl)propan-1-one* (**5b**). Yield: 56%; ^1^H-NMR (300 MHz, CDCl_3_) (ppm): 2.35 (s, 3H), 2.97–3.07 (m, 2H), 3.19–3.29 (m, 2H), 3.68 (s, 2H), 7.13 (dd, 1H), 7.29–7.40 (m, 5H), 7.64 (dd, 1H), 7.73 (dd, 1H); LC-MS: Purity 85%; RT 3.59 min. MH^+^ 260.08 [44].

*3-(Piperidin-1-yl)-1-(thiophen-2-yl)propan-1-one* (**5d**). Yield: 30%; ^1^H-NMR (300 MHz, CDCl_3_) (ppm): 1.33–1.61 (m, 2H), 1.70 (m, 4H), 2.41–2.69 (m, 4H), 2.94 (t, 2H), 3.27 (m, 2H), 7.01–7.19 (m, 1H), 7.63–7.68 (m, 1H), 7.78 (dd, 1H); LC-MS: Purity 83%; RT 2.77 min. MH^+^ 224.13 [42].

*3-[(3,4-Dimethoxybenzyl)(methyl)amino]-1-(thiophen-2-yl)propan-1-one* (**5e**). Yield: 25%; ^1^H-NMR (300 MHz, CDCl_3_) (ppm): 2.38 (s, 3H), 2.97–3.05 (m, 2H), 3.13–3.25 (m, 2H), 3.75 (s, 3H), 3.87–3.91 (m, 5H), 6.61–6.89 (m, 3H), 7.15 (t, 1H), 7.62 (d, 1H), 7.73 (d, 1H); LC-MS: Purity 66%; RT 3.19 min. MH^+^ 320.04.

*3-(Morpholin-4-yl)-1-(thiophen-2-yl)propan-1-one* (**5f**). Yield: 32%; ^1^H-NMR (300 MHz, CDCl_3_) (ppm): 2.63–2.78 (m, 4H), 3.03 (br t, 2H), 3.29 (br t, 2H), 3.78–3.87 (m, 4H), 7.15 (dd, 1H), 7.67 (dd, 1H), 7.79 (dd, 1H); LC-MS: Purity 77%; RT 2.16 min. MH^+^ 226.08 [42].

*3-(4-Benzylpiperidin-1-yl)-1-(5-bromothiophen-2-yl)propan-1-one* (**6a**). Yield: 55%. Yellow oil.; ^1^H-NMR (300 MHz, CDCl_3_) (ppm): 1.55–1.81 (m, 5H), 2.22–2.39 (m, 2H), 2.58 (d, 2H), 2.98–3.10 (m, 2H), 3.11–3.22 (m, 2H), 3.33 (t, 1H), 7.07–7.16 (m, 3H), 7.16–7.26 (m, 2H), 7.26–7.34 (m, 2H), 7.57 (d, 1H); LC-MS: Purity 76%; RT 4.83 min. MH^+^ 391.91 [43].

*3-[Benzyl(methyl)amino]-1-(5-bromothiophen-2-yl)propan-1-one* (**6b**). Yield: 40%. Yellow oil. ^1^H-NMR (300 MHz, CDCl_3_) (ppm): 2.40 (s, 3H), 3.08 (d, 2H), 3.26 (br s, 2H), 3.78 (s, 2H), 7.10 (d, 1H), 7.31–7.44 (m, 5H), 7.50 (d, 1H); LC-MS: Purity 37%; RT 4.20 min. MH^+^ 339.93 [45].

*1-(5-Bromothiophen-2-yl)-3-(piperidin-1-yl)propan-1-one* (**6d**). Yield: 29%; ^1^H-NMR (300 MHz, CDCl_3_) (ppm): 0.80–0.94 (m, 1H), 1.49–1.55 (m, 1H), 1.67–1.80 (m, 4H), 2.57–2.68 (m, 4H), 2.96 (t, 2H), 3.24 (br t, 2H), 7.10–7.13 (m, 1H), 7.54 (d, 1H); LC-MS: Purity 57%; RT 3.61 min. MH^+^ 303.97 [45].

*1-(5-Bromothiophen-2-yl)-3-(morpholin-4-yl)propan-1-one* (**6f**)**.** Yield: 6%; ^1^H-NMR (300 MHz, CDCl_3_) (ppm): 2.62–2.89 (m, 4H), 2.99–3.11 (m, 1H), 3.22–3.34 (m, 1H), 3.55–3.67 (m, 3H), 3.86 (br t, 3H), 7.13 (d, 1H), 7.54 (d, 1H); LC-MS: Purity 38%; RT 3.38 min. MH^+^ 305.91.

## 4. Conclusions

In summary, we have developed a rapid and easy-to-use microwave-assisted protocol based on a combination of PEG 400/CAN, PASPS, and SPE, obtaining the desired products faster than conventional procedures. The reaction optimized with respect to various parameters afforded most of the desired products with good yield and satisfying purity. Our approach could be adapted to a new library of compounds with different aromatic ketones. No less important, the obtained compounds could serve as key intermediates for further functionalization at the ketone group to allow scaffold modifications, suitable for disclosing novel potential hit compounds. We believe that simple reaction procedures and substrate compatibility along with environmentally friendly conditions make our protocol an important supplement to the existing methods.

Lastly, the small focused library we present aims at discovering new potential sigma 1 receptor modulators as part of our ongoing research in this field. From this consideration came our decision to add these products to the library of MuTaLig, an innovative ligand identification platform for the drug-discovery process.

## Figures and Tables

**Table 1:** Investigation of stoichiometric ratio of the reagents on conversion and purity of compound **1a**.

Entry	1 (eq.)	a (eq.)	% Conversion	% Purity
1	1	1	80.0	82.5
2	1	1.5	76.7	77.8
3	1	2	79.9	67.5
4	1	2.5	78.5	60.7
5	1.5	1	67.1	68.9
6	2	1	72.4	97.7
7	2.5	1	77.2	96.1
8	3	1	67.7	95.8

Reagents and reaction conditions: cerium ammonium nitrate (CAN) (0.05 eq.), paraformaldehyde (1.0 eq.), PEG 400, (MW: 90 °C, 60 W, 10 min).

**Table 2:** Investigation of stoichiometric ratio of the reagents on conversion and purity of compound **1b**.

Entry	1 (eq.)	b (eq.)	% Conversion	% Purity
9	1	1	80.0	60.1
10	1	2	77.2	35.1
11	1	2.5	86.9	34.2
12	1	3	93.4	23.5
13	1	3.5	64.9	23.5
14	1.5	1	81.5	69.4
15	2	1	74.5	81.1
16	3	1	61.6	89.6
17	3.5	1	49.2	92.2

Reagents and reaction conditions: CAN (0.05 eq.), paraformaldehyde (1.0 eq.), PEG 400, (MW: 90 °C, 60 W, 10 min).

**Table 3:**
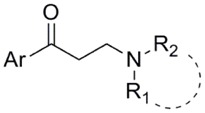
Yield and purity of compounds **1a**–**6f**.

Compound	Ar	NR_1_R_2_	Yield % ^a^	Purity % ^a^
**1a**	phenyl	4-benzylpiperidine	72	98
**1b**		*N*-benzylmethylamine	70	86
**1d**		piperidine	58	75
**1e**		3,4-dimethoxy-*N*-methylbenzylamine	33	66
**1f**		morpholine	-	Traces (5)
**2a**	naphtyl	4-benzylpiperidine	32	69
**2b**		*N*-benzylmethylamine	34	81
**2d**		piperidine	50	75
**2e**		3,4-dimethoxy-*N*-methylbenzylamine	38	54
**2f**		morpholine	46	71
**3a**	biphenyl	4-benzylpiperidine	75	75
**3b**		*N*-benzylmethylamine	55	67
**3d**		piperidine	50	73
**3e**		3,4-dimethoxy-*N*-methylbenzylamine	-	Traces (5)
**3f**		morpholine	50	61
**5a**	2-thienyl	4-benzylpiperidine	63	95
**5b**		*N*-benzylmethylamine	56	85
**5d**		piperidine	30	83
**5e**		3,4-dimethoxy-*N*-methylbenzylamine	25	66
**5f**		morpholine	32	77
**6a**	5-bromo-2-thienyl	4-benzylpiperidine	55	76
**6b**		*N*-benzylmethylamine	40	37
**6d**		piperidine	29	57
**6e**		3,4-dimethoxy-*N*-methylbenzylamine	n.r.	-
**6f**		morpholine	6	38

Reagents and reaction conditions: ketone (2.0 eq.), amine (1.0 eq.), CAN (0.05 eq.), paraformaldehyde (1.0 eq.), PEG 400, (MW: 90 °C, 60 W, 10 min). ^a^ determined by LC-MS analysis. - no data. n.r. no reaction.

## Appendix A

**Table A1:** HPLC analysis for **1a**. XTerra RP18 column (3.5 μm, 4.6 × 50 mm). Flow rate: 1 mL/min.

Time (Minutes)	% Phosphate Buffer	% Acetonitrile
0	90	10
3	90	10
10	60	40
13	60	40
20	5	95
25	90	10
35	90	10

**Table A2:** HPLC analysis for **1b**. Hypersil ODS RP18 column (3 μm, 4.6 × 100 mm). Flow rate: 2 mL/min.

Time (Minutes)	% Phosphate Buffer	% Acetonitrile
0	90	10
3	90	10
10	60	40
13	60	40
20	5	95
30	5	95
35	90	10
40	90	10

## References

[1] Bala S., Sharma N., Kajal A., Kamboj S., Saini V. (2014). Mannich Bases: An Important Pharmacophore in Present Scenario. Int. J. Med. Chem..

[2] Roman G. (2014). Mannich bases in medicinal chemistry and drug design. Eur. J. Med. Chem..

[3] Kalluraya B., Chimbalkar R.M., Hegde J.C. (2005). Anticonvulsant activity of nicotinyl/isonicotinyl substituted 1,2,4-triazol-5-thione Mannich bases. Indian J. Heterocycl. Chem..

[4] Köksal M., Gökhan N., Küpeli E., Yesilada E., Erdogan H. (2007). Analgesic and antiinflammatory activities of some new Mannich bases of 5-nitro-2-benzoxazolinones. Arch. Pharm. Res..

[5] Ashok M., Holla B.S., Poojary B. (2007). Convenient one pot synthesis and antimicrobial evaluation of some new Mannich bases carrying 4-methylthiobenzyl moiety. Eur. J. Med. Chem..

[6] Pandeya S.N., Sriram D., Nath G., De Clercq E. (2000). Synthesis, antibacterial, antifungal and anti-HIV activities of norfloxacin Mannich bases. Eur. J. Med. Chem..

[7] Edwards M.L., Ritter H.W., Stemerick D.M., Stewart K.T. (1983). Mannich bases of 4-phenyl-3-buten-2-one: A new class of antiherpes agent. J. Med. Chem..

[8] Singh B.N., Shukla S.K., Singh M. (2007). Synthesis and biological activity of sulphadiazine Schiff’s bases of isatin and their N-mannich bases. Asian J. Chem..

[9] Malinka W., Świątek P., Filipek B., Sapa J., Jezierska A., Koll A. (2005). Synthesis, analgesic activity and computational study of new isothiazolopyridines of Mannich base type. Farmaco.

[10] Gul H.I., Vepsalainen J., Gul M., Erciyas E., Hanninen O. (2000). Cytotoxic activities of mono and bis Mannich bases derived from acetophenone against Renca and Jurkat cells. Pharm. Acta Helv..

[11] Vashishtha S.C., Zello G.A., Nienaber K.H., Balzarini J., De Clercq E., Stables J.P., Dimmock J.R. (2004). Cytotoxic and anticonvulsant aryloxyaryl Mannich bases and related compounds. Eur. J. Med. Chem..

[12] Ivanova Y., Momekov G., Petrov O., Karaivanova M., Kalcheva V. (2007). Cytotoxic Mannich bases of 6-(3-aryl-2-propenoyl)-2(3*H*)-benzoxazolones. Eur. J. Med. Chem..

[13] Zhang Z., Zhu Y., Zhou C., Liu Q., Lu H., Ge Y., Wang M. (2014). Development of β-amino-carbonyl compounds as androgen receptor antagonists. Acta Pharmacol. Sin..

[14] Lelais G., Seebach D. (2004). β^2^-amino acids-syntheses, occurrence in natural products, and components of β-peptides^1,2^. Biopolymers.

[15] Seebach D., Beck A.K., Bierbaum D.J. (2004). The World of Beta- and Gamma-Peptides Comprised of Homologated Proteinogenic Amino Acids and Other Components. Chem. Biodivers..

[16] Seebach D., Matthews J.L.J. (1997). β-Peptides: A surprise at every turn. Chem. Commun..

[17] Suzuki K., Nagao K., Monnai Y., Yagi A., Uyeda M. (1998). Topostatin, a Novel Inhibitor of Topoisomerases I and II Produced by Thermomonospora alba Strain No. 1520 III. Inhibitory Properties. J. Antibiot..

[18] Ji J.-X., Qiu L.-Q., Yip C.W., Chan A.S.C. (2003). A convenient, one-step synthesis of optically active tertiary aminonaphthol and its applications in the highly enantioselective alkenylations of aldehydes. J. Org. Chem..

[19] Huang P.-J.J., Youssef D., Cameron T.S., Jha A. (2008). Microwave-assisted synthesis of novel 2-naphthol bis-Mannich Bases. Arkivoc.

[20] Azizi N., Baghi R., Ghafuri H., Boloutchian M., Hashemi M. (2010). Silicon Tetrachloride Catalyzed Aza-Michael Addition of Amines to Conjugated Alkenes under Solvent-Free Conditions. Synlett.

[21] Wu J., Zhao C., Wang J. (2016). Enantioselective Intermolecular Enamide−Aldehyde Cross-Coupling Catalyzed by Chiral N-Heterocyclic Carbenes. J. Am. Chem. Soc..

[22] Ye Z., Dai M. (2015). An Umpolung Strategy for the Synthesis of β-Aminoketones via Copper-Catalyzed Electrophilic Amination of Cyclopropanols. Org. Lett..

[23] Cheng J., Qi X., Li M., Chen P., Liu G. (2015). Palladium-Catalyzed Intermolecular Aminocarbonylation of Alkenes: Efficient Access of β-Amino Acid Derivatives. J. Am. Chem. Soc..

[24] Mancuso V., Hootelé C. (1988). A new efficient synthesis of β-aminoketones via Δ^4^-isoxazolines. Tetrahedron Lett..

[25] Tramontini M. (1973). Advances in the chemistry of Mannich bases. Synthesis.

[26] Arend M., Westermann B., Risch N. (1998). Modern Variants of the Mannich Reaction. Angew. Chem. Int. Ed..

[27] Filho J.F.A., Lemos B.C., de Souza A.S., Pinheiro S., Greco S.J. (2017). Multicomponent Mannich reactions: General aspects, methodologies and applications. Tetrahedron.

[28] List B. (2000). The Direct Catalytic Asymmetric Three-Component Mannich Reaction. J. Am. Chem. Soc..

[29] Rossi D., Rui M., Di Giacomo M., Schepmann D., Wünsch B., Monteleone S., Liedl K.R., Collina S. (2017). Gaining in pan-affinity towards sigma 1 and sigma 2 receptors. SAR studies on arylalkylamines. Bioorg. Med. Chem..

[30] Marra A., Rossi D., Pignataro L., Bigogno C., Canta A., Oggioni N., Malacrida A., Corbo M., Cavaletti G., Peviani M. (2016). Toward the identification of neuroprotective agents: G-scale synthesis, pharmacokinetic evaluation and CNS distribution of (R)-RC-33, a promising SIGMA1 receptor agonist. Future Med. Chem..

[31] Rui M., Rossi D., Marra A., Paolillo M., Schinelli S., Curti D., Tesei A., Cortesi M., Zamagni A., Laurini E. (2016). Synthesis and biological evaluation of new aryl-alkyl(alkenyl)-4-benzylpiperidines, novel Sigma Receptor (SR) modulators, as potential anticancer-agents. Eur. J. Med. Chem..

[32] Rossi D., Urbano M., Pedralia A., Serra M., Zampieri D., Mamolo M.G., Laggner C., Zanette C., Florio C., Schepmann D. (2010). Design, synthesis and SAR analysis of novel selective σ1 ligands (Part 2). Bioorg. Med. Chem..

[33] Cobos E.J., del Pozo E., Baeyens J.M. (2007). Irreversible blockade of sigma-1 receptors by haloperidol and its metabolites in guinea pig brain and SH-SY5Y human neuroblastoma cells. J. Neurochem..

[34] Albayrak Y., Hashimoto K. (2017). Sigma-1 Receptor Agonists and Their Clinical Implications in Neuropsychiatric Disorders. Adv. Exp. Med. Biol..

[35] Blicke F.F. (1942). Mannich Reaction. Org. React..

[36] Azzolina O., Collina S., Urbano M., Fata E., Loddo G., Linati L., Lanza E., Barbieri A. (2006). Highly diastereoselective synthesis of enantiopure naphthylaminoalcohols with analgesic properties. Chirality.

[37] Lehmann F., Pilotti Å., Luthman K. (2003). Efficient large scale microwave assisted Mannich reactions using substituted acetophenones. Mol. Divers..

[38] Kabalka G.W., Zhou L.L., Wang L., Pagni R.M. (2006). A microwave-enhanced, solventless Mannich condensation of terminal alkynes and secondary amines with para-formaldehyde on cuprous iodide doped alumina. Tetrahedron.

[39] Kidwai M., Bhatnagar D., Mishra N.K., Bansal V. (2008). CAN catalyzed synthesis of b-amino carbonyl compounds via Mannich reaction in PEG. Catal. Commun..

[40] Flynn G.A., Lee S.A., Faris M., Brandt D.W., Chakravarty S. (2007). Preparation of Aryl Ketone Derivatives as Intracellular Kinase Inhibitors. U.S. Patent.

[41] Jie X., Shang Y., Zhang X., Su W. (2016). Cu-Catalyzed Sequential Dehydrogenation-Conjugate Addition for β-Functionalization of Saturated Ketones: Scope and Mechanism. J. Am. Chem. Soc..

[42] Istanbullu H., Erzurumlu Y., Kirmizibayrak P.B., Erciyas E. (2014). Evaluation of Alkylating and Intercalating Properties of Mannich Bases for Cytotoxic Activity. Lett. Drug Des. Discov..

[43] Debernardis J.F., Kerkman D.J., Zinkowski R.P. (2001). Preparation of Substituted (1-aryl-3-piperazin-1′-yl)Propanone Antibiotics, Antimycotics and Antineoplastics. U.S. Patent.

[44] Yamada T., Sakamoto K., Watanabe K., Nakano Y. (2006). Process for the Preparation of 1-(2-Thienyl)-3-alkylaminopropanols. U.S. Patent.

[45] Mikoshiba K., Hamada K., Terauchi A., Ozaki S., Goto J.I., Ebisui E., Suzuki A. (2011). Transglutaminase-Induced Abnormal Protein Crosslinking Inhibitors Containing Ketone Compounds and Use Thereof. U.S. Patent.

